# Disease and Illness: Prevention, Treatment, Caring, and Health

**Published:** 2011-10-15

**Authors:** Robert J. Ursano

**Affiliations:** Center for the Study of Traumatic Stress, Uniformed Services University

Disease is neither the starting point nor the end point of illness. It is a pathological process that may not be discovered until decades after the identification of an illness. Pathologists are the experts in “disease.” Patients have illness. The disease process may have little obvious connection to the treatment for a patient. For example, strep throat has never been thought of as a penicillin deficiency, yet patients can imagine, just as insulin replaces a deficiency, perhaps penicillin may do the same.

What defines a disease? In the article by Tsai et al (1), nicotine dependence is highlighted as an important and often overlooked disease of veterans. Certainly, the administrative records of the US Department of Veterans Affairs health system underestimate the prevalence of nicotine dependence, but even so, the risk factors identified by Tsai et al improve our understanding of possible prevention and cessation interventions. Mental illness, substance abuse, and homelessness are major problems for which targeted interventions may reduce nicotine dependence. We also know that in the face of disasters — and war is just one type of disaster — smoking increases, further supporting that stress and nicotine use are closely tied (2,3). In fact, post disasters as well as after stressful encounters such as combat, smoking cessation interventions may be one of the best ways to identify both those who may benefit from smoking cessation programs and those with posttraumatic stress disorder (PTSD).

Nicotine dependence and chronic diseases are “illnesses” because they require treatment in a particular person. Treatment targets the disorder, the symptoms, the impairments in physical and psychosocial functioning, disabilities, comorbidities, and the trajectory of the illness. Each of these is a target for both prevention and treatment. Only by addressing all of these areas is an illness treated.

Health risk behaviors — such as smoking — are a particularly important target for treatment and medical intervention. Such interventions must address all stages of the disease and illness and include treatment, prevention, and caring (4). For example, asking for help is a behavior necessary for seeking care. Teaching soldiers how to ask for help and encouraging family members to intervene on their behalf can bring a disease to medical attention before it becomes a chronic illness. Similarly, teaching prevention behaviors such as not smoking or wearing a seatbelt can prevent diseases such as nicotine dependence/addiction (aka smoking) and PTSD, which is many times more likely from injuries sustained in a motor vehicle accident.

The trajectory of illness is a target for treatment and intervention in itself. Preventing chronicity, anticipating relapse, and changing interventions in the recovery stage versus the onset stage are all processes of considering the trajectory in a treatment and prevention plan. Targeting the trajectory of a disorder for intervention — for example, multiple sclerosis, myocardial infarction, depression, or smoking — means being aware of the difference between symptoms in the early-onset phase, mid phase, and chronic phase of the illness. It also means recognizing the predictors of these phases and adapting treatment to the phases, including a transient illness, a relapsing illness, or a chronic illness, all of which may be present in a single patient over time. The importance of treatment and prevention strategies in the recovery and rehabilitation phases of illness and disease is often forgotten in modern medicine; we send the patient home or fail to arrange follow-up care when the illness appears to be under control. The phases of the disease each have specific pathology that is important for intervention and prevention.

Let’s consider a broken arm. Perhaps the broken arm is the second injury. The first was a bruise when the 8-year-old fell out of the tree, playing while his parents were away. It was only with the second fall, when he had climbed even higher, that he broke his arm. If he got to medical care, the bone may have been set, healed well with recovery and restoration of function. But if not, perhaps he hid the injury for several days because of shame and embarrassment, the bone did not set well. An injury has become a chronic impairment and perhaps a disability. The injury was preventable 1) by educating parents about attending to activities of their children even when they are away, 2) early detection of a bruise, 3) educating parents about shame and embarrassment in children who wish to please, or 4) educating the young boy how to manage shame and embarrassment so it does not affect his seeking care.

Example too simple? Apply the same to myocardial infarction, beginning with mild chest pain that was ignored. Or smoking, followed by cough, blood in the sputum, and a positive x-ray.

Our treatments must span the course of disease and illness and must precede the onset to gain opportunities for universal, selective, and targeted interventions for primary, secondary, and tertiary prevention (5).

So let’s return to veterans and nicotine addiction. Rates of smoking increase with combat exposure (6). Depression, PTSD, and other psychiatric disorders are closely linked to smoking. We now have further information from Tsai et al that homelessness is also a risk factor. Screening for PTSD and depression after combat exposure and programs to facilitate employment and prevent homelessness are thus well supported for future trials to reduce nicotine addiction. Such programs are part of treating, preventing, and caring.
